# Improving T cell killing and understanding senescence: Possible roles for *TP53* in cancer immunotherapy

**DOI:** 10.1073/pnas.2402533121

**Published:** 2024-03-11

**Authors:** Arnold J. Levine

**Affiliations:** ^a^Simons Center for Systems Biology, Institute for Advanced Study, Princeton, NJ 08540

At the start of the 21st century, a new understanding of how to use the immune system to detect and kill selective cancers of humans was developed using two different approaches. Checkpoint therapies utilize antibodies directed against proteins on the cell surface that resulted in eliminating or reducing the tolerance of the adaptive immune systems’ ability to kill some solid tumors. Second, CAR-T cells were manufactured by using gene therapies inserting functional receptors that killed several types of leukemias and lymphomas. Remarkably, these approaches resulted in cancer cures, but in most cases, the frequency of curing patients over long time periods was about 20 to 25%. A large number of ways to increase this number have been explored and found (mismatch repair mutations), but by and large, this has been a major roadblock for the field. Many studies of why this is the case suggest poor efficiency of CD-8 killer T cells in repetitive killing of a tumor and/or poor presentation of the tumor antigen to the killer T cell. The reasons why killer T cells, which can be, after all, immortal cells over our lifetime, become exhausted remains an active area of investigation. The manuscript by Roselle et al. (1), in this issue of PNAS, explores a path that appears to be able to reinvigorate the killing by CAR-T cells, and at the same time might well have uncovered an interesting role for the *TP53* gene in regulating T cell biology.

The *TP53* gene is a tumor suppressor that is mutated in about 50 percent of human cancers or is inactivated by other means in many additional neoplasms (2). The wild-type p53 protein is a transcription factor that can regulate hundreds of genes and respond to a wide variety of stresses in cells resulting in either repair and restoring homeostasis or death of the stressed or damaged cells (2). The protein is composed of 393 amino acids divided into five domains: 1. transcription activators, 2. a proline-rich domain, 3. a sequence-specific DNA binding domain, 4. a tetramerization domain, and 5. a regulatory domain (2). The gene generates at least 11 spliced isoforms giving rise to proteins that are combinations of these domain structures (3). This level of complexity seems to detect a very large number of stress signals that disrupt cell cycle regulation, DNA replication and repair, ribosome biogenesis, chromosome segregation, metabolic regulation, glucose utilization, mitochondrial functions, interactions with the immune system (4) and several infectious diseases, and more. It appears that the p53 protein has evolved to integrate a variety of exogenous and endogenous stress signals in cells of an organism and respond by repair and restoration of homeostasis or in a more catastrophic situation respond by instituting one of several programs of cell death at its disposal (2).

The manuscript by Roselle et al. (1), in this issue of PNAS, brings together the laboratories of Carl June (CAR-T cells) and Curt Harris (p53 isoforms) to explore and uncover yet another interesting role for a p53 protein isoform, Δ133p53α (5–7), for its role in CAR-T cells where it can function along with the full-length p53α in response to stresses, as CAR-T cells attempt the killing of a CD-19 leukemia in vitro and in NOD-scid gamma mice. The Δ133p53α isoform is missing the first 132 amino acids from the N terminus of p53. That eliminates the two transactivation signals (at amino acids 25, 26 and 53, 54), the proline domain (at amino acids 55 to 100) (8) deleting the binding sites of the MDM-2 ubiquitin ligase of p53 (at amino acids 25, 26 and 72, 73) (8), and the first 30 amino acids of the DNA-binding domain (the L-1 loop of that domain). It is less clear how this alters or compromises DNA sequence–specific binding and alters transcription. Amino acid residues 133 to 300, fold into a DNA binding domain, but without the L-1 loop. This isoform is compromised in some transcription (no L1 loop of the transactivation domain) and should be compromised in its degradation by MDM-2, leading to high concentrations of Δ133p53α in the cell, with no MDM-2 binding sites but will form tetramers with the wild-type p53 protein because it has a tetramerization domain. The authors recognize all this and so suggest that the Δ133p53α subunits form a mixed tetramer with wild-type protein in the cell resulting in some activities of wild-type p53α and some activities from Δ133p53α mixtures. They test this combined full-length p53 wild type mixed with Δ133p53α isoform in CAR-T cells both in vitro and in vivo (NOD/SCID mice with the NAIM6 Xenograft). In these experiments, the Δ133p53α effector cells cleared the tumor by killing cells more efficiently than T cells with full-length wild-type p53α. Remarkably, the efficiency of tumor clearing in vitro (cell culture) increased as the effector (T cell) to target (tumor cell) ratio decreased from 1:2.5 to 1:10 in cell culture. Similarly, in vivo, mice with larger tumors had their tumor cells killed more efficiently than smaller tumors (determined with tumor luminescence) as the ratio of effector to tumor decreased. The killing of more cells by lower numbers of effectors increases stress upon those T cells, as the T cells undergo more rapid divisions. The p53 protein responds to stresses of various kinds (DNA damage during replication or in resting cells) consistent with the observations for a role of the p53 isoform acting under a stressful condition (2).

The results hold promise for an enhanced efficiency of CAR-T cell production in vitro and CAR-T cell killing of chronic lymphocytic leukemia cells.

The authors explored the genes and mechanisms that the Δ133p53α changed when it was expressed in large amounts in the CAR-T cells using bulk RNA sequencing before and during the co-culturing of T cells with the Δ133-isomer and the Naim6-GFP tumor cells (1:10 effector to target ratio). When the Δ133p53α T cells and full-length p53α T cells were cultured separately, without target cells, there was almost no difference in the RNAs produced. However, when these tumor cells were co-cultured with either Δ133p53α T cells or, separately, with full-length p53α T cells at 10 target-to-T cell ratios, only the Δ133p53α T cells showed 99 out of 100 genes that were down-regulated and these genes were p53 regulated (inducible) genes. There were low levels of p53-regulated m-RNAs from p53-regulated genes, demonstrating the absence of a transactivation domain and a dominant negative impact of Δ133p53α upon the p53 tetramer that forms in the cell. The presence of the Δ133 isomer in a T cell shuts down the induction of p53-regulated genes, which tend to be negative regulators of growth and division. The up-regulated gene sets in the T cells with the Δ133p53α isomer demonstrated biosynthetic processes, enhanced processes of metabolism, cellular integrity, ribosome biogenesis, mitochondrial functions, and translation and telomere maintenance. Some transcription factors were elevated (PGC1α, which enhances mitochondrial functions), as well as protein chromatin modifiers (SIRT-3, a mitochondrial activity), and enriched glucose transport (GLUT-3). A seahorse extracellular flux analysis of the CAR-T cells with or without the Δ133p53α isoform demonstrated increased spare respiratory capacity when the isoform was present. This is consistent with the enhanced expression of PGC1α functions of increased energy production at high energy demands (high levels of tumor killing and CAR-T cell division).

Although the Δ133p53α isomer appears to reduce many of the negative growth functions regulated by p53 wild type, it does not block the abilities of the p53 gene to transcribe its p53-induced DNA repair genes and it contributes to genome stability (5–7). This may be a particularly important contribution to T cell efficiency and viability that is enhanced by Δ133p53α. After killing a target cell, the T cell replicates at a very rapid rate with a very short or no G-1 phase of the cell cycle. The accumulation of DNA replication damage and reactive oxygen discharge needs to be repaired and homeostasis restored.

To begin the process of using the Δ133p53α isoform with chronic lymphocytic leukemia patients who previously failed to benefit from CAR-T therapy, the stored CAR T cells from one patient responding to the treatment and two non-responding patients were employed to produce T cells with Δ133p53α compared to patient T cells with only wild-type full-length p53. The presence of Δ133p53α enhanced the production of the non-responding patients’ T cells in culture, and these CAR-T cells killed at high tumor to effector levels in both the in vitro assay and the in vivo (mice) assay. The T cells from the responder were altered much less with the Δ133p53α than the CAR-T cells from the non-responders with the isoform.

Using a number of different cell types, previous studies with the Δ133p53α isoform have demonstrated an increased induction of several DNA repair systems in the cells, increased uptake and utilization of glucose, increased mitochondrial efficiency, as well as enhanced cellular proliferation (3, 5–7). In normal T cells obtained from healthy individuals, the expression of the Δ133p53α isoform decreases with age of the individual, perhaps contributing to senescense or exhaustion in T cells of the elderly. Further, the addition of Δ133p53α to near-senescent (CD-28,CD57+) T cells enhances the proliferation of these cells (9). The observations made in this manuscript are therefore consistent with and add considerable support to those observations made previously.

The mechanisms of how Δ133p53α acts in a T cell to overcome T cell exhaustion or senescence remain to be elucidated. The isoform protein appears to act as a dominant negative protein for some p53 inducible functions that negatively regulate cell division and permit the expression of the DNA repair and proliferative functions. The isoform could form heterodimers or tetramers with p53, p63, or p73, which have some overlapping sets of gene regulation. Additionally, evidence for mutant p53 or p53 isoforms forming heterodimers or oligomers with other transcription factors leading to new activities in a cell has also been reported (10, 11) and in the case of mutant p53 termed gain of function activities.

This manuscript poses a number of questions in the path to understanding T cell vitality. What is the nature of the stress that activates Δ133p53α CAR-T cells at high target-to-T cell ratios? Is it rapid cell divisions and/or DNA repair? How does the Δ133p53 isomer shut off the induction of the transcription of genes that negatively regulate cell division by p53? How does the full-length p53 protein cause or participate in promoting senescence and cell death in stressed T cells? Could this kind of information (the presence or absence of Δ133p53α) be used to predict responders and non-responders in checkpoint therapy or in CAR-T cell therapy? What is the chance that turning on p53 activities that promote cell division in T cells, and even the killing of tumors, could lead to a malignant T cell phenotype? What additional types of epigenetic or genetic alterations might be needed to bring this T cell transformation about in the presence or absence of a p53 isoform?

Whatever the answers to those questions are, this manuscript identifies roles for the *Tp53* gene, proteins, and isoforms in the immune response, which can now be explored for their role in immunotherapy. The results hold promise for an enhanced efficiency of CAR-T cell production in vitro and CAR-T cell killing of chronic lymphocytic leukemia cells, which for some reason has not been efficient in these patients.
